# Reperfusion times of ST-Segment elevation myocardial infarction in hospitals

**DOI:** 10.12669/pjms.306.5696

**Published:** 2014

**Authors:** Shujuan Dong, Yingjie Chu, Haibo Zhang, Yuhang Wang, Xianzhi Yang, Lei Yang, Long Chen, Haijia Yu

**Affiliations:** 1 Shujuan Dong, Emergency Department of Henan Province People's Hospital, Zhengzhou 450003, China.; 2 Yingjie Chu, Emergency Department of Henan Province People's Hospital, Zhengzhou 450003, China.; 3 Haibo Zhang, Emergency Department of Henan Province People's Hospital, Zhengzhou 450003, China.; 4 Yuhang Wang, Emergency Department of Henan Province People's Hospital, Zhengzhou 450003, China.; 5 Xianzhi Yang, Emergency Department of Henan Province People's Hospital, Zhengzhou 450003, China.; 6 Lei Yang, Emergency Department of Henan Province People's Hospital, Zhengzhou 450003, China.; 7 Long Chen, Emergency Department of Henan Province People's Hospital, Zhengzhou 450003, China.; 8 Haijia Yu, Emergency Department of Henan Province People's Hospital, Zhengzhou 450003, China.

**Keywords:** Henan Province, ST-segment elevation myocardial infarction, Reperfusion time, Study

## Abstract

***Objective: ***To investigate the reperfusion time in patients with ST-segment elevation myocardial infarction (STEMI) in Henan Province, China, and discuss the strategies for shortening that period.

***Methods: ***The reperfusion times of 1556 STEMI cases in 30 hospitals in Henan Province were analyzed from January 2008 to August 2012, including 736 cases from provincial hospitals, 462 cases from municipal hospitals and 358 cases from country hospitals. The following data: Time period 1 (from symptom onset to first medical contact), Time period 2 (from first medical contact to diagnosis), Time period 3 (from the diagnosis to providing consent), Time period 4 (from the time of providing consent to the beginning of treatment) and Time period 5 (from the beginning of treatment to the patency) were recorded and analyzed.

***Results:*** In patients receiving primary percutaneous coronary intervention, the door-to-balloon time of provincial hospitals and municipal hospitals was 172±13 minutes and 251±14 minutes, respectively. The hospitals at both levels had a delay comparison of 90 minutes largely caused by the delay in the time for obtaining consent. In patients receiving thrombolysis treatment, the door-to-needle times of provincial hospitals, municipal hospitals and country hospitals were 86±7, 91±7 and 123±11 minutes, respectively. The hospitals at all levels had delays lasting more than 30 minutes, which was mainly attributed to the delay in the time for providing consent. Compared with the time required by the guidelines, the reperfusion time of patients with STEMI in China is evidently delayed. In terms of China's national conditions, the door-to-balloon time is too general. Therefore, we suggest refining this time as the first medical contact–diagnosis time, consent provision time, therapy preparation time and the start of therapy balloon time.

***Conclusion:*** Compared to the time required by the guidelines, the reperfusion time of patients with STEMI in China was obviously greater. In terms of China's national conditions, the door to balloon time is not applicable. So it is suggested to refine it as the first medical contact-diagnosis time, providing consent time, therapy prepare time and the start of therapy – balloon time.

## INTRODUCTION

The World Health Organisation reported in 2011 that about 30% of worldwide deaths were caused by cardiovascular diseases. Compared with past data, rates of death from cardiovascular diseases have declined in most developed countries because of improved awareness and timely treatment. By contrast, people in developing countries are more prone to cardiovascular diseases because of various reasons. ST-segment elevation myocardial infarction (STEMI) has high morbidity and mortality as a common and severe presentation of cardiovascular diseases. Annually, more than 3 million people suffer or die from STEMI worldwide.^1^ Restoring coronary flow and reperfusing myocardial tissue^2^^,^^3^ as early as possible is currently proven as the key link to the treatment of STEMI.^4^^,^^5^ For these patients, saving time is saving life. Fast transport and timely treatment are both paramount. However, reperfusion delay has become an urgent worldwide problem to be solved, especially for developing countries.

Retrospective studies^6^^-^^8^ show that the factors associated with longer time to treat STEMI vary in different areas. In China, reperfusion delay has existed for long time, but large-scale research in this area is scarce. Among the several known studies, non-systematic reasons are prevalent^9^^,^^10^ unlike in other countries. This manuscript aims to analyze this issue, mainly about in-hospital delay, to explore a solution suitable for China.

## METHODS

***Clinical Data: ***We surveyed the primary percutaneous coronary intervention times of 1556 STEMI cases (complete data) of 30 provincial hospitals, municipal hospitals and country hospitals in Henan Province from January 2008 to August 2012. Provincial hospitals have one to four catheter laboratories, and municipal hospitals have a single catheter laboratory. The patients all underwent routine examinations, such as ECG, cardiac enzymes, troponin, coagulation function, blood routine, kidney function, electrolytes and blood sugar. The patients had no statistical significance in age, sex, smoking, hypertension, diabetes and dyslipidaemia. According to the hospitals approached by the patients, the 1556 cases were stratified into three subgroups: (1) 736 cases from provincial hospitals with 589 males and 147 females all aged (56±6); (2) 462 cases from municipal hospitals with 370 males and 92 females all aged (57±6); and (3) 358 cases from country hospitals with 289 males and 69 females all aged (60±7). STEMI diagnosis was in accordance with the naming and diagnostic criteria of the Chinese Medical Association on STEMI.^11^ Exclusion criteria included the patients who did not receive primary percutaneous coronary intervention or had received failed intervention. This study was conducted in accordance with the declaration of Helsinki after approval from the Ethics Committee of Henan Province People's Hospital. Written informed consent was obtained from all participants.

***Research Methods: ***We analyzed the reperfusion times of 1556 STEMI cases of 30 hospitals, including 736 cases from provincial hospitals, 462 cases from municipal hospitals and 358 cases from country hospitals. We set the boundaries as symptom onset, first medical contact, diagnosis, providing consent, start of therapy (the beginning time of medication in thrombolytic therapy or the vascular puncture time in primary percutaneous coronary intervention) and reperfusion time (the time of meeting the reperfusion standards in thrombolytic therapy or the time of balloon dilatation in primary percutaneous coronary intervention) in each case. We recorded the following: Time period 1 (from symptom onset to first medical contact), Time period 2 [diagnosis time (from first medical contact to diagnosis)], Time period 3 [providing consent time (from the diagnosis to providing consent, including the communication time of the illness state, the time of treatment schedule explanation and the time of decision making)], Time period 4 [treatment preparation time (from the time of providing consent to the beginning of treatment)] and Time period 5 (from the beginning of treatment to the patency). We recorded the correlation time of each case and then analyzed the recorded data.

***Statistical analysis:*** The data were presented as mean±standard deviation (±s), and the means of the groups were compared by ANOVA and q-test. The statistical significance level was p<0.05 (two-sided test). All data were processed by SPSS 13.0 statistical software package.

## RESULTS

***Reperfusion time of primary percutaneous coronary intervention:*** The comparison between Group 1 and Group 2 in Time periods 1, 2 and 3 had no statistical significance (*P*>0.05) (Table-I). Time periods 4 and 5 of Group 1 were significantly shorter than those of Group 2 (*P*<0.05) (Table-II). Group 3 had no primary percutaneous coronary intervention cases. In patients receiving primary percutaneous coronary intervention, the door-to-balloon time of Group 1 was 172±13 minutes and that of Group 2 was 251±14 minutes. The hospitals in both groups took more than 90 minutes, taking too much time to obtain consent and prepare the treatment.

***Revascularisation time of thrombolysis treatment:*** The comparison between Group 1 and Group 2 in the time of symptom onset, first medical contact, diagnosis, providing consent, start of therapy and reperfusion time had no statistical significance. The time of providing consent and the time from symptom onset to first medical contact Group 3 were significantly longer than those of Groups 1 and 2 (*P*<0.05) (Table-III). In patients receiving primary thrombolysis treatment, the door-to-thrombolysis time of Group 1 was 86±7 minutes, that of Group 2 was 91±7 minutes and that of Group 3 was 123±11 minutes. The times of the three groups all exceeded 30 minutes and were mainly spent in obtaining consent.

## DISCUSSION

In the past two decades, the diagnosis and treatment of STEMI has been remarkably enhanced.^11^^,^^12^ The successful launch of primary percutaneous coronary intervention has led to the decrease in STEMI mortality from 30% to less than 5%.^13^^,^^14^ The cardiovascular branch of the Chinese Medical Association developed the 2010 STEMI diagnosis and treatment guidelines based on the ACC/AHA treatment guidelines of STEMI that was updated in 2009,^15^ with the core idea of achieving reperfusion as early as possible.^3^ This strategy requires the emergency department to complete the clinical examination within 10 min for patients with suspected AMI, record the monitor 18-lead ECG (conventional 12 leads plus V7 to V9 and V3R to V5R) and interpret it. Pharmacological reperfusion should be performed within 30 minutes or PCI within 90 minutes. However, the primary percutaneous coronary intervention time of patients with STEMI is evidently delayed.^16^^,^^17^ Recent surveys show that the door-to-balloon time is borderline with regard to the guidelines for only 19% of patients.^2^^,^^3^

**Table-I:** Characteristics, medical history and gender differences among the three groups of patients

	***Group 1 (n=736)***	***Group 2 (n=462)***	***Group 3 (n=358)***	***P***
Age: Years ≤45 46-55 56-65 66-75 ≥76	110 248 297 61 20	78 147 182 40 15	55 120 143 30 10	0.831 0.489 0.109 0.051 0.299
Male sex	589	370	289	0.08
Education: Years ≤10 11-15 ≥16	236 377 123	150 244 68	121 190 47	0.334 0.457 2.556
On social security	406	260	202	0.220
Medical history: hypertension diabetes mellitus dyslipidaemia Previously diagnosed angina Positive family history	290 98 354 329 177	180 61 234 201 116	134 51 183 164 84	0.400 0.227 1.196 0.439 0.322

**Table-II:** Revascularization time distribution of primary percutaneous coronary intervention ( ±s).

***Group***	***n***	***Time period1***	***Time period2***	***Time period3***	***Time period4***	***Time period5***
1	612	133±11	10±1	58±10	72±8	28±5
2	246	141±13	11±1	61±11	126±11*	51±7*
3	0	0	0	0	0	0

***Note:*** Comparisons with the Group 1 were

* *P*<0.05.

**Table-III:** Revascularization time distribution of thrombolysis treatment ( ±s).

***Group***	***n***	***Time period1***	***Time period2***	***Time period3***	***Time period4***	***Time period5***
1	124	134±9	10±1	8±10	19±5	57±6
2	216	138±11	10±1	61±12	22±7	58±8
3	358	173±15*	12±1	89±15*	25±7	62±7

***Note:*** Comparisons with the Group 1 and Group 2 were

* *P*<0.05.

The economic and medical levels of Henan Province, which has a population of over a hundred million, are domestic mid-stream so the primary revascularization time of Henan Province can reflect the overall situation in China. Unlike other studies, this manuscript is an accurate reflection of the actual situation in the country. In this study, we set the boundaries as symptom onset, first medical contact, diagnosis, providing consent, start of therapy (the beginning time of medication in thrombolytic therapy or the vascular puncture time in emergency percutaneous coronary intervention) and reperfusion time (the time of meeting the reperfusion standards in thrombolytic therapy or the time of balloon dilatation in emergency percutaneous coronary intervention) and recorded the time from symptom onset to first medical contact, the time of diagnosis, the time of providing consent (from the diagnosis to providing consent, including the communication time of illnesses' state, the time of explaining treatment schedules and the time of making decisions), the time of the start of therapy (from providing consent to the start of treatment, which can be divided into the time for patients' family to prepare the surgery cost and medical preparation time) and the time from the start of treatment to the patency time. Among these boundaries, the times for providing consent and treatment preparation are unique in our country.^9^^,^^18^ In addition, we looked at a lot of foreign medical literature in the developing countries, but the related research has not been found.

In terms of Time period 1, the time of patients in treatment in the three groups was visibly longer than that reported in foreign developed countries. The time of patients in treatment in the country hospitals was the longest; this finding indicates the differences in various aspects, such as scientific quality, patients' willingness, medical conditions and creation of an emergency rescue system. Time period 2 met the guideline requirements because of the strong feasibility of the STEMI guidelines. Time period 3 cannot be found in foreign literature and guidelines. However, it is an objective reality in China that has existed for an extended time period, and it restricts the reperfusion time into a shorter period. To solve this problem, China should improve the quality of human science and doctor-patient relationships, extend the healthcare reform and solve the problems in cost of the treatment. Time period 4 can be divided into 4A and 4B. 4A denotes the time period for the patients' families to pay the cost of surgery. No such time period is recorded in foreign literature, but this period is also an objective reality in China and has existed for a long time. Nevertheless, China should not only solve the patients' worry about social health insurance but also effectively implement the system of prioritising who gets treated first. 4B is the time period that involves the medical preparation time, which also does not exist in foreign literature. This period usually delays the reperfusion time considerably^19^ mainly because most hospitals lack 24hour medical staff and the catheter laboratory is occupied. The fundamental solution^20^^,^^21^ is to build catheter laboratories for primary percutaneous coronary intervention and require the personnel to be on duty for 24 hour. In terms of Time period 5, Group 1 recorded a shorter time than Group 2.^22^

In patients received in-hospital fibrinolytic therapy, the delays of providing consent in Group 3 were longer than those in Groups 1 and 2. In addition to the reasons mentioned above, the communication skills of doctors are also an important factor.

Notably, no case of pre-hospital thrombolysis was reported in this group of patients. Moreover, no case of communication about the reperfusion was found during their transfer to the hospitals; this aspect also requires improvement.

***Limitations:*** This study aimed to investigate the reperfusion time of patients with STEMI based on the part of the provincial, municipal and county hospitals in Henan province, to a certain extent, so the result may be affected by the influence of the geographical, economic, cultural and medical resources. Then, it cannot fully represent the overall treatment level of developing countries. In addition, because it is a retrospective study, clinical features of some cases such as height, waist circumference, body mass index were not documented and detail analyzed. Moreover, this study was not undertaken with strict follow-up.

Overall, the primary percutaneous coronary intervention time of STEMI was clearly longer than that in foreign developed countries^23^^,^^24^ and as required in guidelines^9^ because of both patients and hospitals. The former was mainly caused by pre-hospital delay, consent delay and medical cost delay; the latter mainly caused by the lack of 24 hour open catheter laboratory and medical staff, low medical technology level and the unimplemented policy of getting treatment first. This issue must be solved by expanding the healthcare reform and improving the quality of human science, medical technology level and doctor-patient relationship. Therefore, in terms of China's national conditions, the door-to-balloon time is not applicable. We suggest re-defining this time as the first medical contact-diagnosis time, providing consent time, therapy preparation time and start of therapy-balloon time to comply with the actual work in various aspects of the clinical, research, teaching, quality control and policy formulation in this field as well as to meet the needs of this specialty.

## Authors’ contribution:

**Shujuan Dong** **:** Conceived, designed and performed the study; accountable for the study; drafted and revised the manuscript; approved the final version.

**Yingjie Chu:** Conceived and designed the study; accountable for all aspects of the study; revised the manuscript; approved the final version.

**Yuhang Wang and Xianzhi Yang:** Contributed to the conception of the study; approved the final version.

**Haibo Zhang, Lei Yang and Long Chen:** Performed the study; acquired data.

**Haijia Yu:** Acquired data and helped perform the analysis.
